# Indoor CO_2_ monitoring in a surgical intensive care unit under visitation restrictions during the COVID-19 pandemic

**DOI:** 10.3389/fmed.2023.1052452

**Published:** 2023-07-14

**Authors:** Ying-An Chou, Zheng-Yao Wang, Hsiang-Ching Chang, Yi-Chia Liu, Pei-Fang Su, Yen Ta Huang, Chao-Tung Yang, Chao-Han Lai

**Affiliations:** ^1^Department of Surgery, National Cheng Kung University Hospital, College of Medicine, National Cheng Kung University, Tainan, Taiwan; ^2^Department of Computer Science, Tunghai University, Taichung, Taiwan; ^3^UniSmart Technology Co., Ltd., Taichung, Taiwan; ^4^Department of Statistics, College of Management, National Cheng Kung University, Tainan, Taiwan; ^5^Research Center for Smart Sustainable Circular Economy, Tunghai University, Taichung, Taiwan; ^6^Department of Biochemistry and Molecular Biology, College of Medicine, National Cheng Kung University, Tainan, Taiwan; ^7^Department of Biostatistics, Vanderbilt University Medical Center, Nashville, TN, United States

**Keywords:** indoor air quality, carbon dioxide, intensive care unit, visitation restriction, COVID-19 pandemic, indoor environment monitoring system, internet of things

## Abstract

**Background:**

Indoor CO_2_ concentration is an important metric of indoor air quality (IAQ). The dynamic temporal pattern of CO_2_ levels in intensive care units (ICUs), where healthcare providers experience high cognitive load and occupant numbers are frequently changing, has not been comprehensively characterized.

**Objective:**

We attempted to describe the dynamic change in CO_2_ levels in the ICU using an Internet of Things-based (IoT-based) monitoring system. Specifically, given that the COVID-19 pandemic makes hospital visitation restrictions necessary worldwide, this study aimed to appraise the impact of visitation restrictions on CO_2_ levels in the ICU.

**Methods:**

Since February 2020, an IoT-based intelligent indoor environment monitoring system has been implemented in a 24-bed university hospital ICU, which is symmetrically divided into areas A and B. One sensor was placed at the workstation of each area for continuous monitoring. The data of CO_2_ and other pollutants (e.g., PM2.5) measured under standard and restricted visitation policies during the COVID-19 pandemic were retrieved for analysis. Additionally, the CO_2_ levels were compared between workdays and non-working days and between areas A and B.

**Results:**

The median CO_2_ level (interquartile range [IQR]) was 616 (524–682) ppm, and only 979 (0.34%) data points obtained in area A during standard visitation were ≥ 1,000 ppm. The CO_2_ concentrations were significantly lower during restricted visitation (median [IQR]: 576 [556–596] ppm) than during standard visitation (628 [602–663] ppm; *p* < 0.001). The PM2.5 concentrations were significantly lower during restricted visitation (median [IQR]: 1 [0–1] μg/m^3^) than during standard visitation (2 [1–3] μg/m^3^; *p* < 0.001). The daily CO_2_ and PM2.5 levels were relatively low at night and elevated as the occupant number increased during clinical handover and visitation. The CO_2_ concentrations were significantly higher in area A (median [IQR]: 681 [653–712] ppm) than in area B (524 [504–547] ppm; *p* < 0.001). The CO_2_ concentrations were significantly lower on non-working days (median [IQR]: 606 [587–671] ppm) than on workdays (583 [573–600] ppm; *p* < 0.001).

**Conclusion:**

Our study suggests that visitation restrictions during the COVID-19 pandemic may affect CO_2_ levels in the ICU. Implantation of the IoT-based IAQ sensing network system may facilitate the monitoring of indoor CO_2_ levels.

## 1. Introduction

Indoor air quality (IAQ) is a prominent health concern related to the modern lifestyle because people spend approximately 90% of their daily time indoors (1–3). Several harmful pollutants inside buildings may deteriorate IAQ, including carbon dioxide (CO_2_), volatile organic compounds (VOCs), PM2.5, and others (4). Among these pollutants, CO_2_ is a known constituent of the atmosphere and a major metabolite released by humans (4–7). Exposure to a high indoor CO_2_ concentration may produce a variety of health effects. Since indoor CO_2_ concentration has been widely promoted as an important metric of IAQ, many practitioners and researchers use 1,000 ppm as a criterion to define good IAQ. Notably, studies have challenged what was previously considered to be good IAQ. Evidence demonstrates the association between lower levels of indoor CO_2_ (below 1,000 ppm) and sick building syndrome, including perceptions of stuffiness, respiratory symptoms, tiredness, and loss of concentration (6, 8–11). Risks of these non-specific syndromes are elevated when the indoor CO_2_ levels rise. The standard considers CO_2_ a proxy for other indoor air pollutants (12). Moderate concentrations of indoor CO_2_ are associated with changes in human performance and decision-making ability (13). Several standards and guidelines (e.g., the International WELL Building Standard and the United Kingdom Health and Safety Executive) recommend 800 ppm or even a lower level as a threshold for indoor CO_2_ levels in concern of potential risks of adverse health effects and cognitive impairment (14,15). Accordingly, monitoring indoor CO_2_ levels may be important for IAQ control, potentially contributing to the health and performance of occupants (14), especially for those experiencing high levels of cognitive load.

The intensive care unit (ICU) is a specialized ward and one of the most critically functioning operational environments in the hospital. ICUs are also densely populated areas full of patients and healthcare professionals. In ICUs, critically ill patients with limited physiological reserve to tolerate error continuously receive multiple therapeutic procedures 24 hours a day, making the tasks of healthcare providers cognitively demanding and mentally stressful. Thus, the performance of healthcare providers in ICUs may be susceptible to IAQ changes. A number of studies have investigated IAQ in different inpatient and outpatient areas in hospitals (5, 16–31). In most of these studies, however, periodic sampling rather than continuous measurement was performed because frequent air sampling and analysis are costly, labor-intensive, and time-consuming (17, 32). Moreover, only two of these studies focused on indoor CO_2_ levels in ICUs (5, 20). The dynamic temporal pattern of CO_2_ levels in ICUs, where the occupant numbers are frequently changing, has not been comprehensively characterized.

Understanding the dynamic change in CO_2_ levels in ICUs using continuous monitoring may help develop IAQ control strategies to prevent potential threats from CO_2_. In the studies presented here, we attempted to describe the dynamic change in CO_2_ levels in ICUs using an Internet of Things-based (IoT-based) IAQ monitoring system implemented in our surgical ICUs. The entire system combines applications of grid computing and cloud technologies to create an efficient, low-cost, real-time, and lightweight IAQ monitoring network (17). Specifically, given that the COVID-19 pandemic makes hospital visitation restrictions necessary worldwide (33–35), no established data are available regarding the impact of visitation restrictions on CO_2_ levels in ICUs. These findings may provide a basis for the reappraisal of standard visitation policies in the ICU in terms of IAQ control.

## 2. Materials and methods

### 2.1. Study site

**Figure 1:**
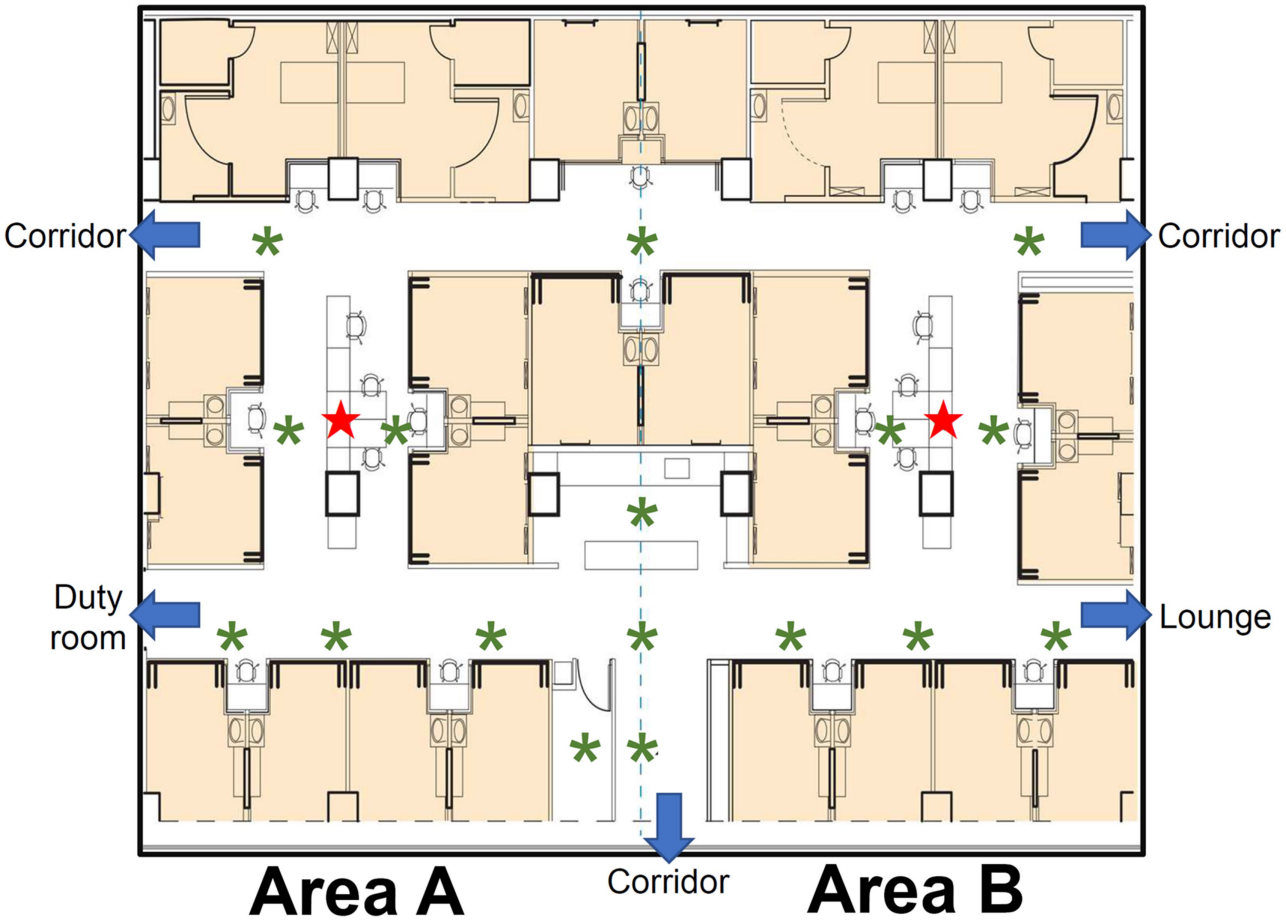
Schematic layout of the study ICU, SICU1. The patient rooms are in light orange background color. The ICU is divided into two symmetric areas, areas A and B. The sensors, indicated by red stars, are placed in the workstation of each area. The inlets of HVAC system are indicated by green asterisks.

The present study was conducted in a large 24-bed surgical intensive care unit (SICU1) of National Cheng Kung University Hospital, a 1,300-bed medical center that offers first-line and tertiary referral services for 1.8 million people in southern Taiwan. This unit, located on the third floor of the main hospital building, provides postoperative care for neurosurgery, general surgery, and traumatology services. The occupancy rate is stable at >95% year-round, even during the COVID-19 pandemic. Figure 1 shows the schematic layout of the SICU1, in which there are no operable windows or openings that access the outdoors. The total floor area is 979.03 m^2^ and height is 2.5 meter. The unit is symmetrically divided into areas A and B, with a workstation and 12 beds in each area. Each bed is in a separate room, and the door is open unless there is a special requirement (e.g., isolation). The workstation serves as a working space where healthcare providers can use desktop computers. Two areas communicate freely without a gate, electric door, or portiere on the hallways. The IAQ of the SICU1 is regulated by central ventilation and air conditioning, which controls the temperature and relative humidity within a narrow band. The Heating, Ventilation, and Air Conditioning (HVAC) system equipped in the SICU1 is 39 K, a central station air handler manufactured by Carrier, Taiwan. This modular design system that has a flexible airflow rate ranging from 100 to 88,235 cube feet per minute (CFM). The final airflow rate employed in the SICU1 is 21,630 CFM; thus, there are 15 air changes per hour. The ventilation was constant during the study period. The HVAC system has been thoroughly evaluated by engineers annually, and the airflow of each area in the SICU1 is presumed to be identical.

### 2.2. IAQ system design and implementation

We have continuously monitored real-time air quality in the ICU since February 2020. Indoor CO_2_ concentration was measured using Plantower PTQS1005, a diffusive, non-dispersive infrared (NDIR) sensor (IAQ-CALC, TSI, USA) capable of measuring up to 5,000 ppm of CO_2_, with a resolution of 1 ppm and tolerance within 50 ppm or 3% of reading. For continuous monitoring, one sensor was placed at the workstation of each area (Figure 1), located at the center of the area and surrounded by the patient rooms. Each sensor was set at a height of 1.1 meters above the floor, and no oxygen supply device or electronic product was allocated around the sensors. Air quality sampling was conducted at 1-min intervals to obtain real-time CO_2_ levels.

**Figure 2:**
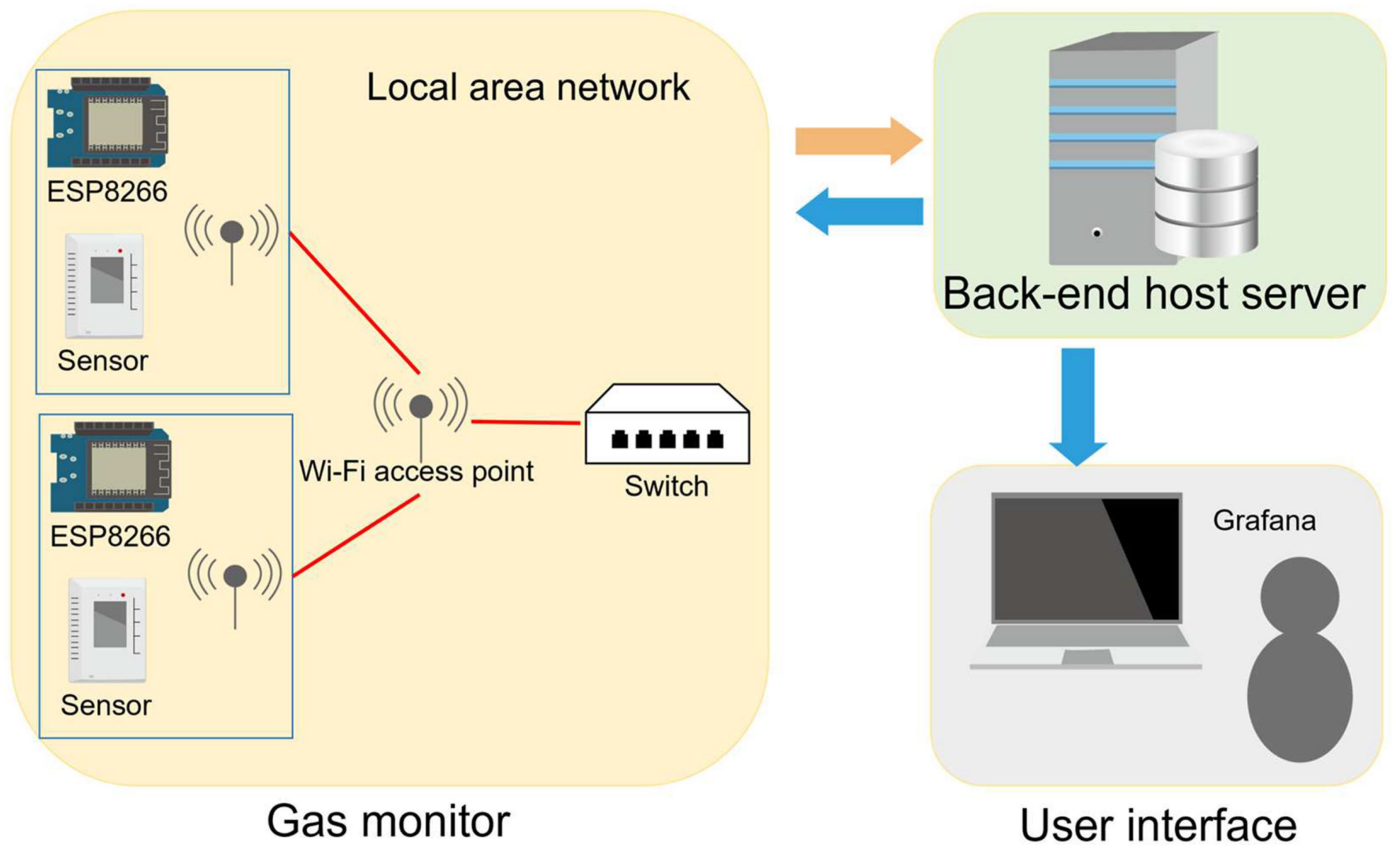
Scenario of web service setup. The indoor environment monitoring system (iEMS) consists of air quality sensors, a host server with a MySQL database and a Grafana graphic user interface (GUI). The ESP8266 micro control unit (MCU) and Wi-Fi module are integrated to connect with the Wi-Fi access point in the air quality sensor. The ESP8266 MCU integrated the Wi-Fi module connected to the Wi-Fi switch for transferring data. When CO_2_ values are sampled by sensors, these data are transmitted to the Wi-Fi switch and sent to the backend of the server via a local area network (LAN). The collected CO_2_ value is visualized with Grafana and can be operated with client equipment in real-time. Once the collected CO_2_ value reaches a default limit, alarm signals are transmitted to the Wi-Fi switch to trigger the front-end of the digital plug.

The indoor environment monitoring system (iEMS) used in the present study is built with sensors. This iEMS consists of some air quality sensors, a host server with a MySQL database and a Grafana graphic user interface (GUI). The ESP8266 micro control unit (MCU) and Wi-Fi module are integrated to connect with the Wi-Fi access point in the air quality sensor (Figure 2). Between the ESP8266 MCU integrated Wi-Fi module and Wi-Fi switch (access point), the IEEE 802.11 b/g/n is used as a standard to transmit data wirelessly. When sensors sample CO_2_ values, the data are transmitted to the Wi-Fi switch and sent to the backend of the server via the local area network (LAN). Once the collected CO_2_ value reaches a default limit, the ESP8266 MCU transmits alarm signals through the Wi-Fi module to the Wi-Fi switch (access point) to trigger the front end of the digital plug. The compiler formulates UART (Universal Asynchronous Receiver/Transmitter) codes that capture the CO_2_ data from sensors. The database environment is set up using Apache Web Server Version 2.4.29, PHP Script Language Version 7.2.24, and MySQL Database Version 5.7.37. The backend host server functions as the data monitoring, analysis, and plug controller. This system is implemented with JSON format for data collection and exchange and transmitted data in JSON or XML format to increase consistency and readability. System data are visualized with Grafana (Version 6.2.5; Grafana Labs, Stockholm, Sweden) for analysis and can be operated with client equipment in real time (17). The CO_2_ data are available to staff in the ICU in a real-time manner. However, we only monitored and collected data without conducting intervention during the study period.

### 2.3. Staffing and visitation policies

Occupants in the unit include patients, healthcare providers, and visitors. Healthcare providers involved in the care for critically ill patients include physicians, nurse practitioners, nurses, dietitians, pharmacists, respiratory therapists, secretaries, maintenance workers, and administration staff. The regular numbers of healthcare providers and other occupants in areas A and B within specified time intervals are summarized in Table 1. During the COVID-19 pandemic, the study unit was normally functioning for the care of surgical patients. Visitation policies were regulated by the administration of the hospital. Under the standard visitation policy, the visiting time was scheduled twice daily, from 10:30 AM to 11:00 AM and 6:00 PM to 6:30 PM. For each patient, two visitors were allowed to enter the SICU1 during visitation. Under the restricted visitation policy, all visitation was prohibited except for special conditions such as patient expiration.

**Table 1:** The staff numbers at each area on workdays and non-working days.

Shift	Staff	Workdays	Non-working days
Morning	Doctor	4	2
	Nurse practitioner	3	1
	Nurse	14	13
	Dietitian	0.5	0
	Pharmacist	1	0
	Respiratory therapist	1	1
	Secretary	1	0
	Maintenance worker	2	2
	Administration staff	1	0.5
	Total	27.5	18.5
Middle	Duty doctors/nurse practitioner	2	2
	Nurse	14	13
	Maintenance worker	0.5	0.5
	Respiratory therapist	0.5	0.5
	Total	17	16
Night	Duty doctor/nurse practitioner	2	2
	Nurse	13	13
	Maintenance worker	0.5	0.5
	Respiratory therapist	0.5	0.5
	Total	16	16

### 2.4. Data retrieval and statistical analysis

To investigate the effect of restricted visitation on CO_2_ levels, data from the first two weeks after the policy change were eliminated as the washout period. Thus, we retrieved data from three surveillance intervals (phase 1 [from April 26, 2020, to May 9, 2020], phase 2 [from October 28, 2020, to December 17, 2020], and phase 3 [from May 30, 2021, to July 3, 2021]). The restricted visitation policy was implemented during phase 1 and phase 3, whereas phase 2 operated under standard visitation. During the rest of the period, a variety of partial visitation restriction policies were conducted, such as restriction on visitor number (e.g., one visitor permitted for each patient), frequency (e.g., once daily), or both. Also, these partial visitation restriction policies were swiftly altered, leading to insufficient washout periods. Thus, we decided to omit the rest of the period. In addition, we analyzed the daily temporal variation in several pollutants (i.e., PM2.5, formaldehyde, and VOCs [excluding formaldehyde]) during restricted visitation versus standard visitation. Given that the occupant numbers are different on workdays and non-working days, we also explored the differences in indoor CO_2_ levels during workdays and non-working days and between areas A and B, and responsible data were compared.

Categorical variables, expressed as numbers and percentages, were analyzed using the χ^2^ test or Fisher’s exact test as needed. Continuous variables, expressed as median and interquartile range (IQR) or mean and standard deviation as appropriate, were compared using the Wilcoxon rank-sum test. Statistical analyses were performed using R software (Version 3.4.3; Foundation for Statistical Computing, Vienna, Austria). A two-tailed *p* value <0.05 was considered statistically significant.

## 3. Results

### 3.1. Summary of descriptive data for indoor CO_2_ levels

**Figure 3:**
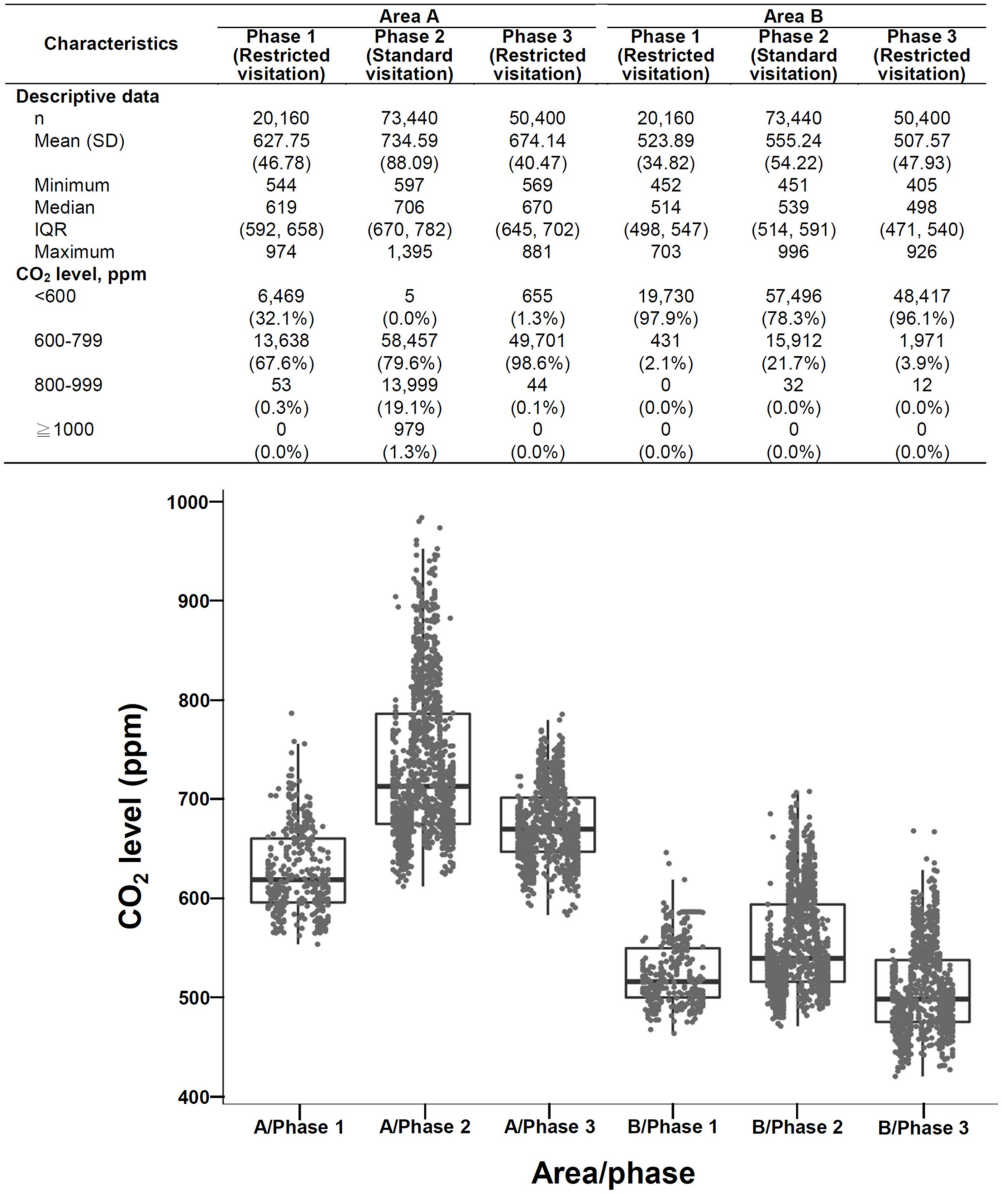
Overview (descriptive data and boxplots) of CO_2_ concentrations during three surveillance intervals area A (left) and area B (right) of SICU1. SD: standard deviation, IQR: interquartile range. Each point in the figure represents the mean value of hourly data.

A total of 288,000 data points were collected during three monitoring intervals. The CO_2_ levels ranged from 405 ppm to 1,395 ppm (Figure 3), and the median and IQR were 616 (524–682) ppm. CO_2_ levels ≥1,000 ppm are scarcely detected. Only 979 (0.34%) data points obtained in area A during phase 2 (standard visitation) were ≥ 1,000 ppm, whereas 132,772 (46.1%) data points were < 600 ppm. The CO_2_ concentrations varied among different phases and areas. The highest and lowest concentrations of CO_2_ were found in area A during phase 2 (median [IQR]: 706 [670–782] ppm) and in area B during phase 3 (median [IQR]: 498 [471–540] ppm), respectively. Across the 3 phases, at least 78.3% of CO_2_ data in area B were < 600 ppm, whereas only 32.1% or less of CO_2_ data in area A were < 600 ppm.

### 3.2. Levels of CO_2_ and other pollutants: restricted visitation versus standard visitation

**Figure 4:**
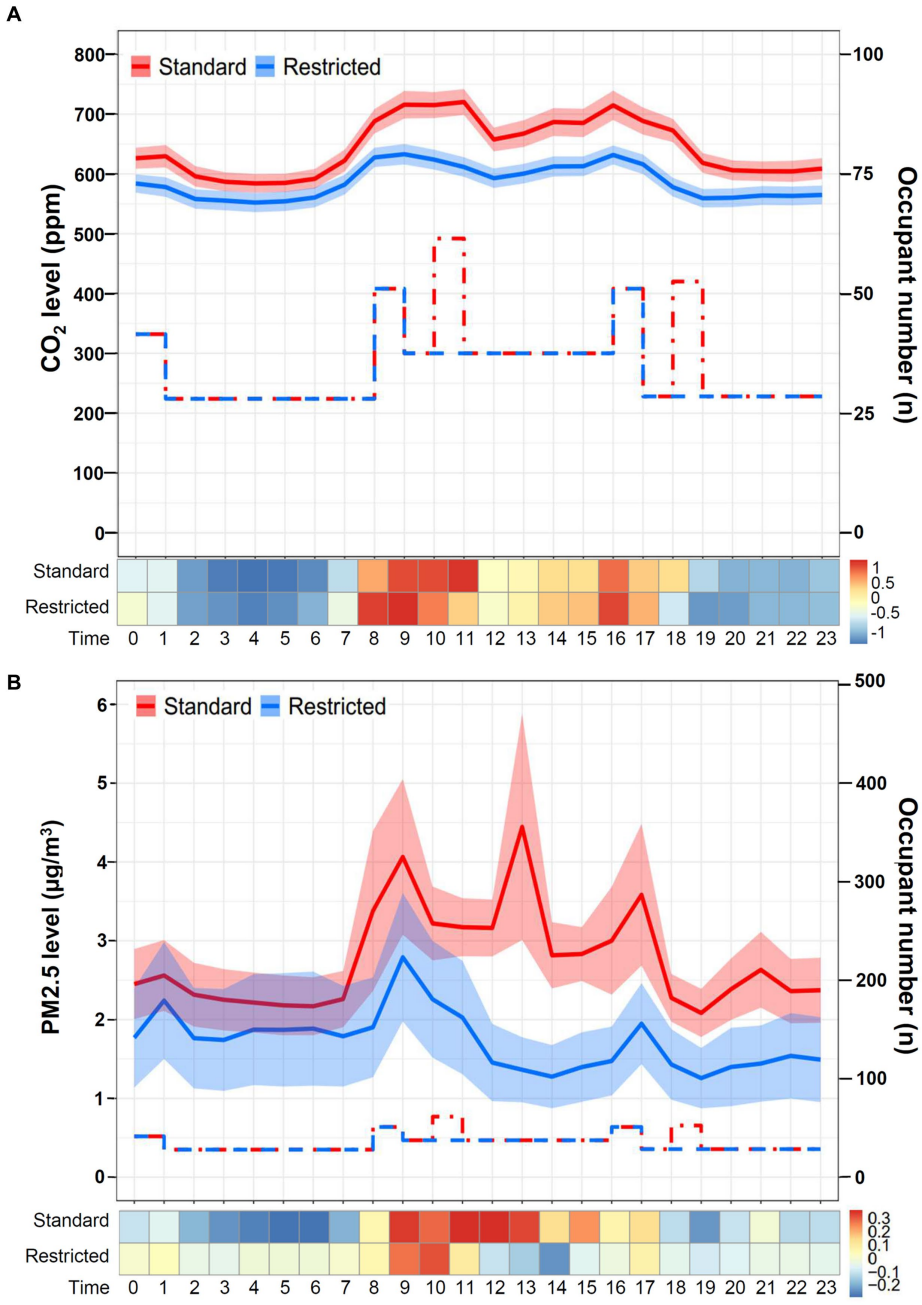
Daily temporal variation in CO_2_ and PM2.5 levels during restricted visitation versus standard visitation. **(A)** The line chart and Z-score heat map depict the change in daily CO_2_ concentrations during restricted visitation versus standard visitation. The line chart demonstrates hourly CO_2_ levels in mean and standard deviation. The step plot represents the estimated hourly occupant numbers during standard visitation (red dot-dash line) and restricted visitation (blue dash line). The Z-score in the heat map is transformed based on the mean and standard deviation in each group. **(B)** The line chart and Z-score heat map depict the change in daily PM2.5 concentrations during restricted visitation versus standard visitation. The line chart demonstrates hourly PM2.5 levels in mean and standard deviation. The step plot represents the estimated hourly occupant numbers during standard visitation (red dot-dash line) and restricted visitation (blue dash line). The Z-score in the heat map is transformed based on the mean and standard deviation in each group.

The daily temporal variation in CO_2_ levels during restricted visitation versus standard visitation is shown in Figure 4A. The CO_2_ concentrations were significantly lower during restricted visitation (phase 1 and phase 3 combined; median [IQR]: 576 [556–596] ppm) than during standard visitation (phase 2; 628 [602–663] ppm; *p* < 0.001). Regardless of visitation policies, the daily CO_2_ level was relatively low at night and elevated during the daytime as the occupant number increased, especially at the time of clinical handover and visitation. The daily temporal variation in PM2.5, formaldehyde, and VOCs (excluding formaldehyde) during restricted visitation versus standard visitation were also analyzed. The PM2.5 concentrations (Figure 4B) were significantly lower during restricted visitation (phase 1 and phase 3 combined; median [IQR]: 1 [0–1] μg/m^3^) than during standard visitation (phase 2; 2 [1–3] μg/m^3^; *p* < 0.001). Regardless of visitation policies, the daily PM2.5 level was low at night and higher during the daytime, especially at the time of clinical handover and visitation. The formaldehyde concentrations (Supplementary Figure S1) were significantly lower during restricted visitation (phase 1 and phase 3 combined; median [IQR]: 34 [26–44] μg/m^3^) than during standard visitation (phase 2; 42 [32–54] μg/m^3^; *p* < 0.001). During restricted and standard visitation, the daily formaldehyde level was relatively low at night and elevated during the daytime as the occupant number increased, especially during clinical handover and visitation. Finally, the VOC concentrations (Supplementary Figure S2) were not different during restricted visitation (phase 1 and phase 3 combined; median [IQR]: 0.017 [0.015–0.021] ppm) than during standard visitation (phase 2; 0.018 [0.015–0.021] ppm; *p* = 0.31). Regardless of visitation policies, the daily VOC level was relatively constant but was slightly elevated during clinical handover.

**Figure 5:**
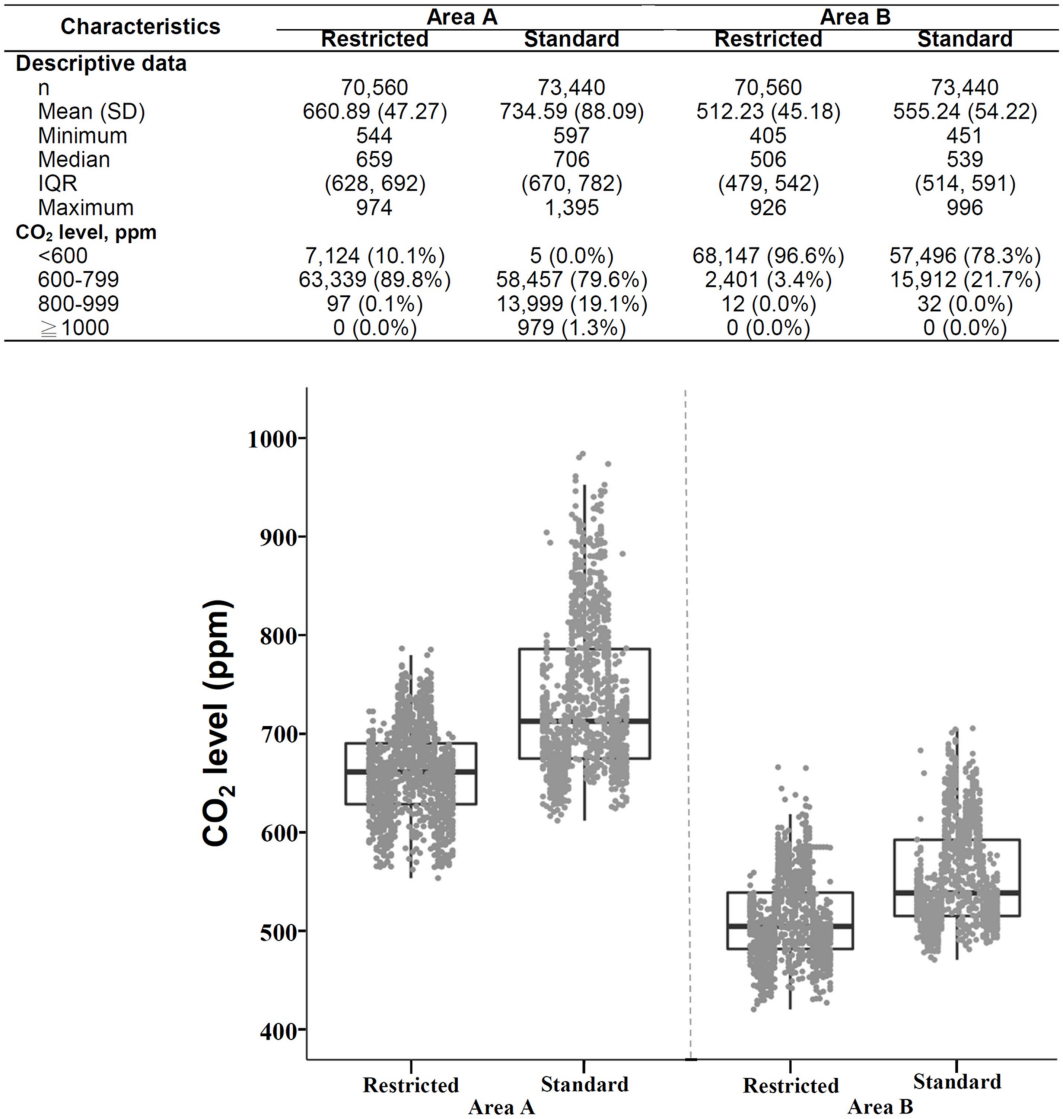
Descriptive data and boxplots of CO_2_ concentrations during restricted visitation versus standard visitation in area A (left) and area B (right) of SICU1. SD, standard deviation; IQR, interquartile range. Each point in the figure represents the mean value of hourly data.

As shown in Figure 5, the CO_2_ levels during restricted visitation (median [IQR]: 659 [628–692] ppm) were significantly lower than those during standard visitation (706 [670–782] ppm, *p* < 0.001) in area A. The overwhelming majority of CO_2_ concentrations during restricted visitation were < 600 ppm (10.1%) and 600–799 ppm (89.8%), whereas the majority during standard visitation were 600–799 ppm (79.6%) and 800–999 ppm (19.1%), indicating a different frequency distribution between restricted and standard visitation (*p* < 0.001). Similarly, the CO_2_ levels during restricted visitations (median [IQR]: 506 [479–542] ppm) were significantly lower than those during standard visitation (539 [514–591] ppm, *p* < 0.001) in area B. The proportions of CO_2_ concentrations <600 ppm were 96.6 and 78.3% during restricted and standard visitation (*p* < 0.001), suggesting that the frequency distribution differed between restricted and standard visitations. As shown in Supplementary Figure S3, the PM2.5 levels during restricted visitation (median [IQR]: 1 [0–1] ppm) were significantly lower than those during standard visitation (2 [1–4] ppm, *p* < 0.001) in area A. Likely, the PM2.5 levels during restricted visitations (median [IQR]: 1 [0–2] ppm) were significantly lower than those during standard visitation (2 [1–3] ppm, *p* < 0.001) in area B.

### 3.3. CO_2_ levels: area A versus area B

**Figure 6:**
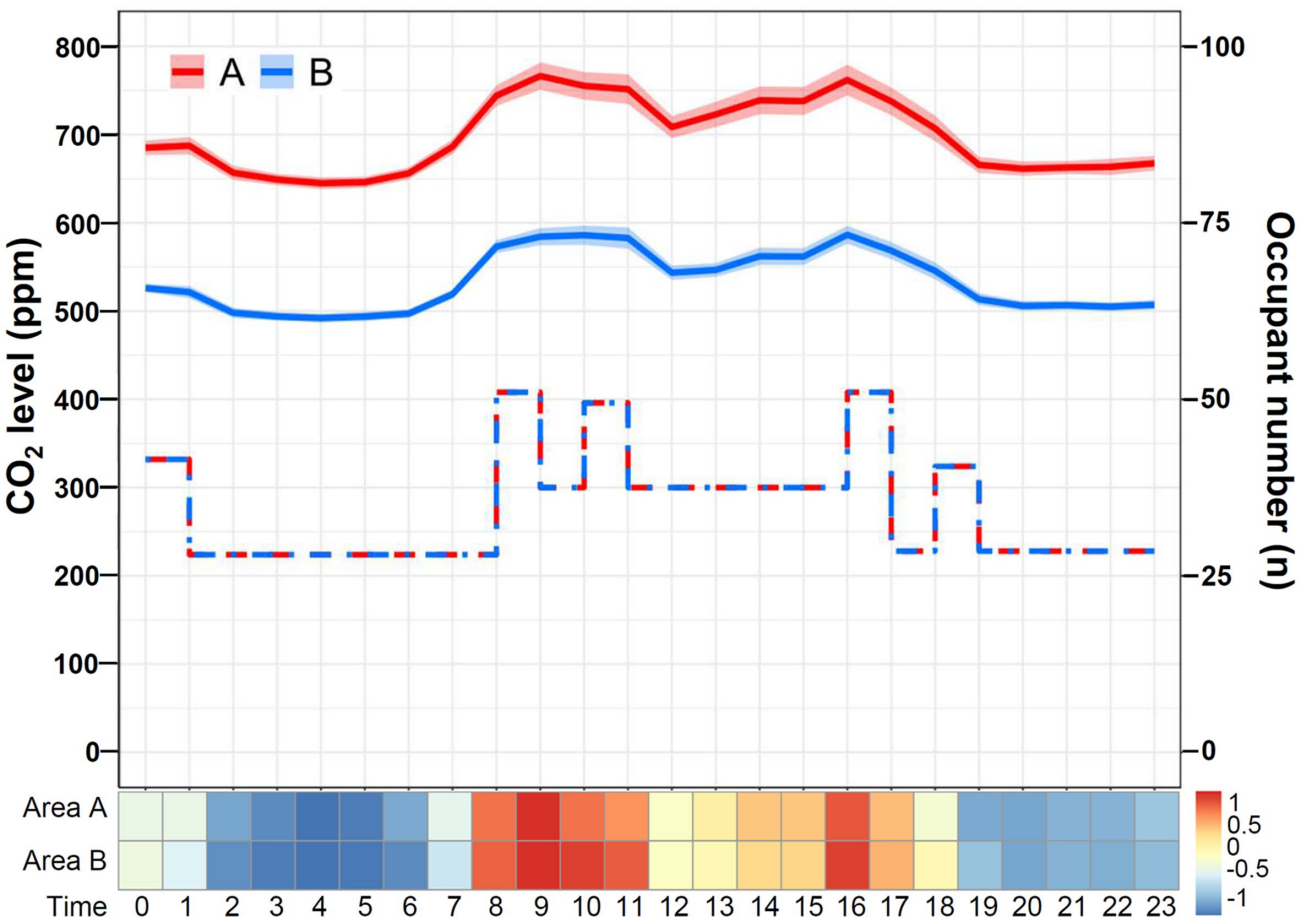
Daily temporal variation in CO_2_ levels in area A versus area B of SICU1. The line chart and Z-score heat map depict the change in daily CO_2_ concentrations in areas A and B. The line chart demonstrates hourly CO_2_ levels in mean and standard deviation. The step plot represents the estimated hourly occupant numbers at areas A (red dash line) and B (blue dot-dash line). The Z-score in the heat map is transformed based on the mean and standard deviation in each group.

The daily temporal variation in CO_2_ levels in area A versus area B of the SICU1 is shown in Figure 6. Although the sensors were both placed in the SICU1, the CO_2_ concentrations recorded were significantly higher in area A (median [IQR]: 681 [653–712] ppm) than in area B (524 [504–547] ppm; *p* < 0.001). For both area A and area B, the CO_2_ level was low at night and elevated during the day, compatible with the expected diurnal change in occupancy patterns. During restricted visitation (Figure 5), the CO_2_ levels were significantly higher in area A (median [IQR]: 659 [628–692] ppm) than in area B (506 [479–542] ppm, *p* < 0.001). The frequency distribution of CO_2_ concentrations was different in area A or area B (*p* < 0.001); the majority in area A were 600–799 ppm (89.8%), whereas the majority in area B were < 600 ppm (96.6%). Similarly, under the standard visitation policy, the CO_2_ levels were significantly higher in area A (median [IQR]: 706 [670–782] ppm) than in area B (539 [514–591] ppm, *p* < 0.001). The overwhelming majority of CO_2_ concentrations in area A were 600–799 ppm (79.6%) and 800–999 ppm (19.1%), whereas almost all in area B were < 600 ppm (78.3%) and 600–799 ppm (21.7%), suggesting a different distribution between area A and area B (*p* < 0.001).

### 3.4. CO_2_ levels: non-working days versus workdays

**Figure 7:**
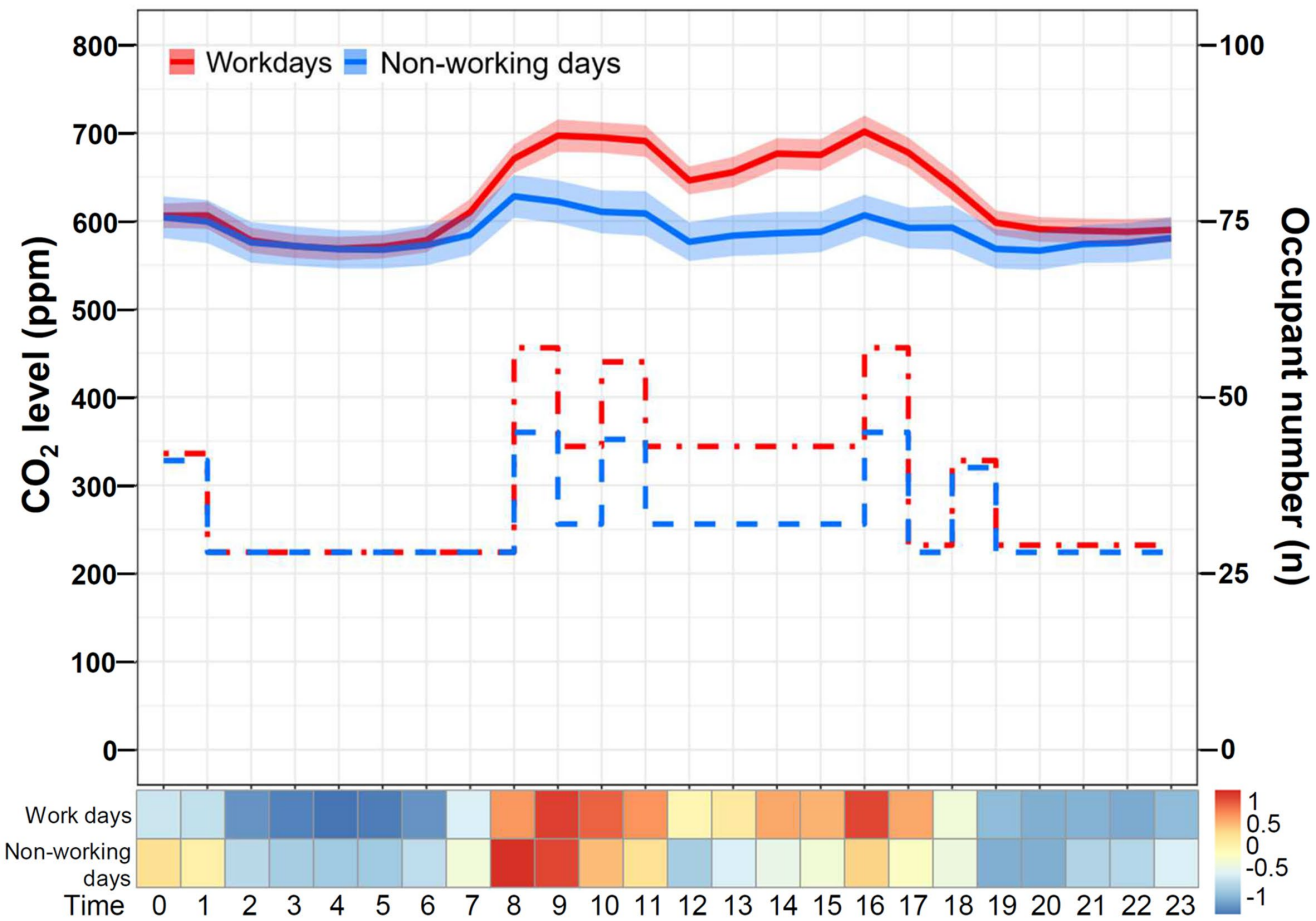
Daily temporal variation in CO_2_ levels on workdays and non-working days. The line chart and Z-score heat map depict the change in daily CO_2_ concentrations on workdays and non-working days. The line chart demonstrates hourly CO_2_ levels in mean and standard deviation. The step plot represents the estimated hourly occupant numbers on workdays (red dot-dash line) and non-working days (blue dash line). The Z-score in the heat map is transformed based on the mean and standard deviation in each group.

**Figure 8:**
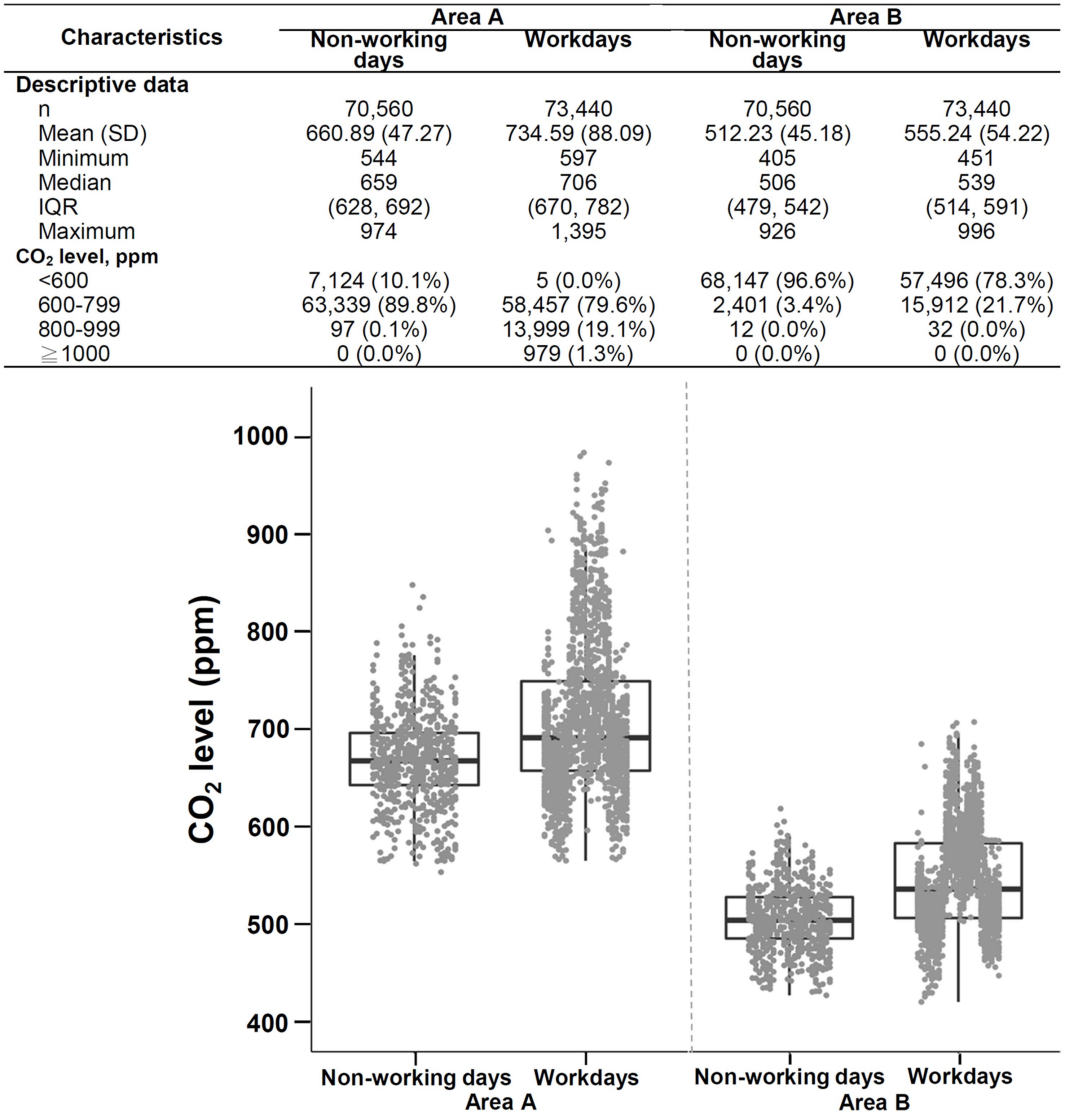
Descriptive data and boxplots of CO_2_ concentrations during non-working days versus workdays in area A (left) and area B (right) of SICU1. SD, standard deviation; IQR, interquartile range. Each point in the figure represents the mean value of hourly data.

The daily temporal variation in CO_2_ levels on non-working days versus workdays is shown in Figure 7. The CO_2_ concentrations were significantly lower on non-working days (median [IQR]: 606 [587–671] ppm) than on workdays (583 [573–600] ppm; *p* < 0.001). On workdays, the daily CO_2_ level declined at night and increased during the daytime. Likely, a similar pattern was observed on non-working days, although the variation appeared relatively minor. Notably, the difference existed during the daytime as the occupant number increased, compatible with the difference in the morning shift between workdays and non-working days (Table 1). In contrast, the CO_2_ concentrations overnight (11:00 PM to 7:00 AM) on workdays and non-working days were not different (*p* = 0.72). As shown in Figure 8, the CO_2_ levels on non-working days (median [IQR]: 659 [628–692] ppm) were significantly lower than those on workdays (706 [670–782] ppm, *p* < 0.001) in area A. Additionally, the CO_2_ levels on non-working days (median [IQR]: 506 [479–542] ppm) were significantly lower than those on workdays (539 [514–591] ppm, *p* < 0.001) in area B.

## 4. Discussion

### 4.1. Principal findings

The features and trends of IAQ may differ significantly in different working areas in medical institutions (22, 28, 29, 36, 37). During the COVID-19 pandemic, we investigated the impact of visitation policies on indoor CO_2_ levels in the ICU, where people work around the clock, yet the occupant number is highly dynamic (32). We found that the daily CO_2_ level corresponded with expected diurnal occupancy patterns: lower overnight and higher during the day. The indoor CO_2_ levels were significantly higher under the standard visitation policy than under the restricted visitation policy, suggesting that visitation restriction policies during the COVID-19 pandemic period may pose an impact on CO_2_ levels in the ICU. The CO_2_ levels were significantly higher in area A than in area B, even though both were in the same unit. Additionally, the levels on non-working days were lower than those on workdays, consistent with the notion that higher occupant density leads to CO_2_ accumulation. The indoor environment monitoring system may facilitate monitoring the dynamic change in indoor CO_2_ levels.

### 4.2. Strengths

The COVID-19 pandemic is a catastrophe that has led to a dramatic loss of human lives worldwide and has presented an unprecedented economic and social disruption (38). Although Taiwan was estimated to be most influenced by COVID-19 due to its proximity to mainland China, the outbreak in Taiwan has been controlled well under an effective public health strategy (39). Visitation restrictions have been implemented in healthcare facilities during the COVID-19 pandemic, providing a valuable opportunity to demonstrate the effect of visitation policies on indoor CO_2_ levels.

In the present study, we used an IoT-based IAQ system to monitor and record indoor CO_2_ concentrations. IAQ may be associated with poor productivity and various occupational damage in medical practitioners (40). Therefore, IAQ monitoring has gradually become crucial in hospital management. However, no consensus has been established regarding the approach of monitoring IAQ in the hospital. Thus, developing an intelligent, reliable, and cost-effective sensing network system that possesses functions such as sensing and monitoring IAQ becomes imperative (17). Manual air sampling is cost-intensive and may not provide real-time data (17, 32), hardly reflecting the dynamic changes in indoor CO_2_ levels (19–21, 25, 28, 31, 40, 41). Our system combines applications of grid computing and cloud technologies to create an efficient, low-cost, and real-time IAQ control network (17, 42). This system serves as a platform for data analysis, file access, and transmission, facilitating the storage and analysis of data collected from sensors.

### 4.3. Comparison with previous literature

Only two of them have focused on indoor CO_2_ levels in ICUs (5, 20). Tang et al. conducted indoor air sampling in a 4-bed patient room in the medical ICU on a fixed weekly day for 1 year (20). The duration of each sampling was 90 min, including 30 min before patient visitation, 30 min during patient visitation, and 30 min after patient visitation. Notably, most CO_2_ samples (92%) exceeded the recommended indoor limit of 1,000 ppm. The values of indoor CO_2_ were higher after visitation than before visitation. Interestingly, an increased number of patient visitors was not related to the increased indoor CO_2_ concentrations. Licina et al. performed IAQ monitoring in a neonatal ICU during a 1-year study period (5). The CO_2_ levels and presence or absence of occupants were measured continuously during workdays with a 1-min resolution. Indoor CO_2_ levels were within the range typical of well-ventilated indoor environments (~500 ppm) and showed moderate variability. No association between CO_2_ levels and local occupancy in individual baby rooms was demonstrated. The authors proposed that CO_2_ emissions anywhere in the ICU would propagate evenly by recirculating airflow in the HVAC system.

### 4.4. Interpretation

In contrast, the present study demonstrated that CO_2_ levels differed even in the same unit with the same HVAC system. Indoor CO_2_ levels may be affected by ventilation rates, occupant activity levels, or outdoor air quality (16, 21, 23, 43). Without more information on the specific areas measured, it is not easy to speculate on the reasons for the differences in CO_2_ levels observed. However, it is possible that ventilation could play a role in the observed differences. Accordingly, performing a more detailed inspection and maintenance to ensure optimal performance of the HVAC system and avoid potentially poor airflow in area A may be necessary. Another explanation may be the different activities of occupants (21). This speculation cannot be confirmed as the information about the type and intensity of their activities performed were not evaluated.

More critical, visitation policies may contribute to the difference in indoor CO_2_ levels. Because CO_2_ elevation is mainly a consequence of metabolic CO_2_ generation by occupants (4–7), visitation policy modification to control the number of visitors might be a considerable intervention to improve IAQ (19–21, 31). This approach might be supported by lower CO_2_ levels on non-working days than on workdays. Nevertheless, restricted visitation may result in psychological distress for patients and their families (33). Additionally, physician-family interactions are essential in critical care. Thus, suspension of ICU visitation as a routine measure to improve IAQ may not be feasible. In addition, given the spatial variations and wide variability in ICU visitation policies in different hospitals (44, 45), introducing an efficient IAQ surveillance program using a technologically mature, cost-effective real-time CO_2_ detection system appears more practical. Real-time CO_2_ levels represent the interactions between the efficacy of the air-conditioning system and the dynamics of occupancy number and other possible sources. Administrators can monitor real-time IAQ at the designated areas through the fast-response system and notify medical staff as the CO_2_ level deteriorates. While awareness of the problem is of utmost importance (46), IAQ can be maintained by achievement of adequate ventilation or diversion of visitor inflows in a reactive manner. This concept may also be applied to PM2.5 control, given that the concentrations of CO_2_ and PM, the important IAQ indices, are correlated with the number of persons in a space (18, 25).

The similar daily temporal pattern between CO_2_ and PM2.5 and their correlation with occupancy patterns suggest that the two pollutants are correlated, compatible with the observation shown in a recent study by Butler et al. (47). Activation of air filtration can lower the risk of exposure to respiratory pathogens (48). Given that respirable particulate matter (e.g., PM2.5) is made up partly of bioaerosol that contains pathogens (23), improving ventilation under IAQ surveillance may play a role in mitigating the threat of disease transmission, particularly for patients cared for in the ICU.

### 4.5. Limitations

The present findings must be interpreted within the context of the study limitations. First, traditional patient outcomes in ICU settings, such as mortality and length of stay, and the performance of healthcare providers, whose loads are cognitively challenging, were not evaluated in this study. No conclusion can be achieved regarding the effect of indoor CO_2_ levels on these aspects. Thus, further studies are warranted. Second, the study was conducted in only one ICU, and the design was descriptive rather than controlled. The information regarding occupant numbers was estimated based on the regular staff numbers (Table 1) and visitors (i.e., two visitors permitted for each patient during standard visitation) rather than obtained through real-time direct-field observation. Additionally, the information about the type and intensity of their activities performed was not recorded. Indoor CO_2_ levels may be affected by a variety of factors, such as ventilation rates, occupant activity levels, outdoor CO_2_ levels, proximity to areas with high traffic or industrial activity, or even wind direction (16, 21, 23, 43); thus, the findings may not be extrapolated directly to other medical facilities. Finally, implementing an IoT-based monitoring system requires the installation of sensors, data collection devices, and network infrastructure. Additionally, the system needs to be properly maintained and updated to ensure reliable and accurate data collection. These setups can be complex, and thus expertise in IoT technologies is required. These considerations may affect the generalizability of the findings and study approaches. Validation of the findings shown in this study in other ICUs is highly warranted.

### 4.6. Conclusion

In conclusion, using an IoT-based IAQ sensing network system, our data suggested that visitation restrictions during the COVID-19 pandemic may affect CO_2_ levels in the ICU. Implantation of the IAQ sensing network system may facilitate the monitoring of indoor CO_2_ levels.

## Data availability statement

The original contributions presented in the study are included in the article/Supplementary material, further inquiries can be directed to the corresponding authors.

## Author contributions

C-HL is the guarantor of this article. Y-AC, C-TY, and C-HL: study conception and design. Z-YW and H-CC: acquisition of data. Y-AC, Y-CL, P-FS, YH, and C-HL: analysis and interpretation of data. Y-AC, Z-YW, H-CC, Y-CL, P-FS, YH, C-TY, and C-HL: manuscript drafting and critical review. All authors contributed to the article and approved the submitted version.

## Funding

This work is supported by grants from the Ministry of Science and Technology, Executive Yuan, Taiwan (MOST 110-2326-B-006-002 and MOST 111-2314-B-006-018-MY3 to C-HL).

## Conflict of interest

Z-YW and H-CC were employed by UniSmart Technology Co., Ltd.

The remaining authors declare that the research was conducted in the absence of any commercial or financial relationships that could be construed as a potential conflict of interest.

## Publisher’s note

All claims expressed in this article are solely those of the authors and do not necessarily represent those of their affiliated organizations, or those of the publisher, the editors and the reviewers. Any product that may be evaluated in this article, or claim that may be made by its manufacturer, is not guaranteed or endorsed by the publisher.
