# Supplementary material for: Indoor CO2 monitoring in a surgical intensive care unit under visitation restrictions during the COVID-19 pandemic
Source: Front Med (Lausanne). 2023 Jul 14;10:1052452. doi: 10.3389/fmed.2023.1052452 (PMC10375033; doi:10.3389/fmed.2023.1052452)
Supplement: Supplementary file 1 [file Data_Sheet_1.PDF]

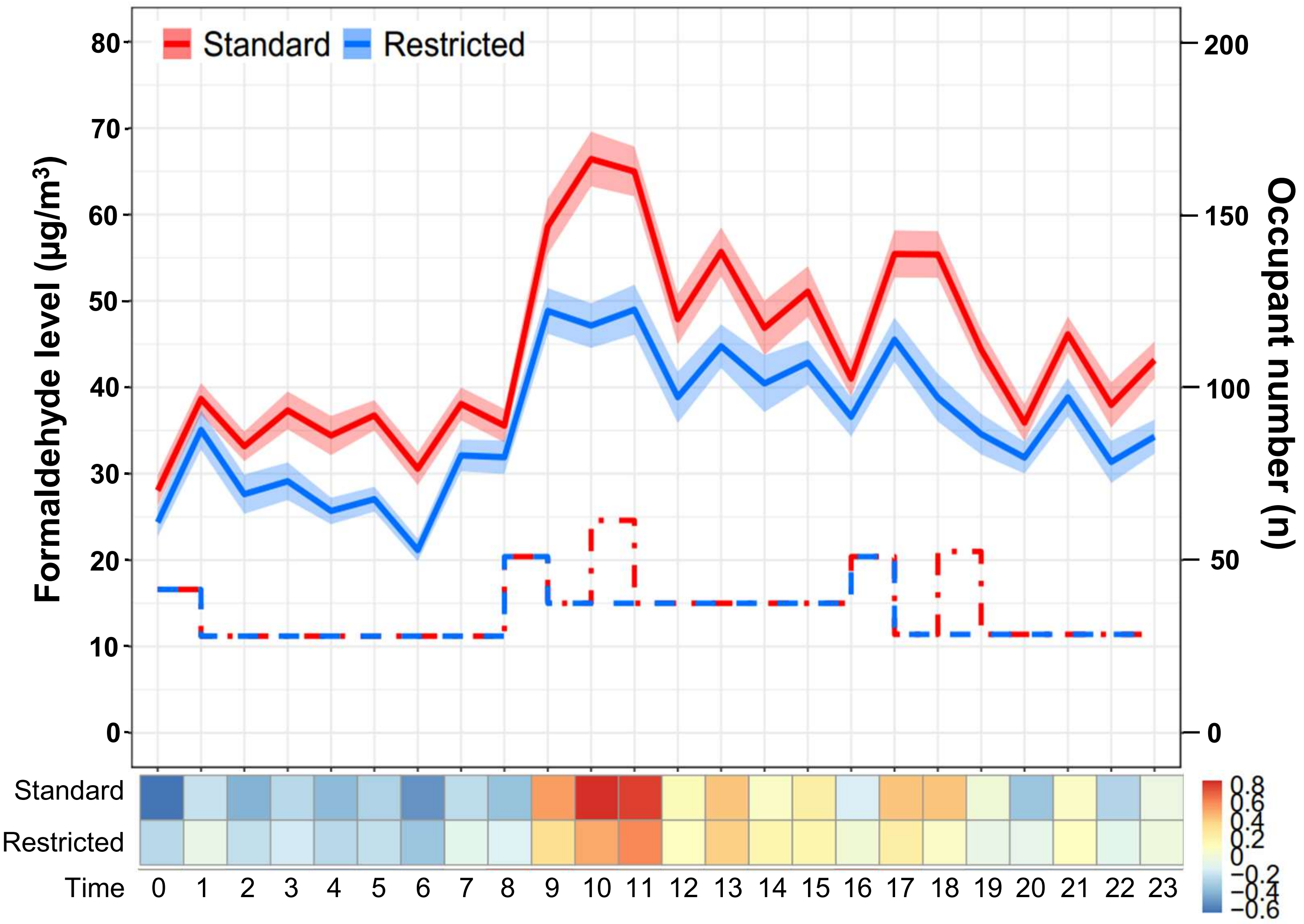

Supplementary Figure 1. Daily temporal variation in formaldehyde levels during restricted visitation versus standard visitation. The line chart and Z-score heat map depict the change in daily formaldehyde concentrations during restricted visitation versus standard visitation. The line chart demonstrates hourly formaldehyde levels in mean and standard deviation. The step plot represents the estimated hourly occupant numbers during standard visitation (red dot-dash line) and restricted visitation (blue dash line). The Z-score in the heat map is transformed based on the mean and standard deviation in each group.

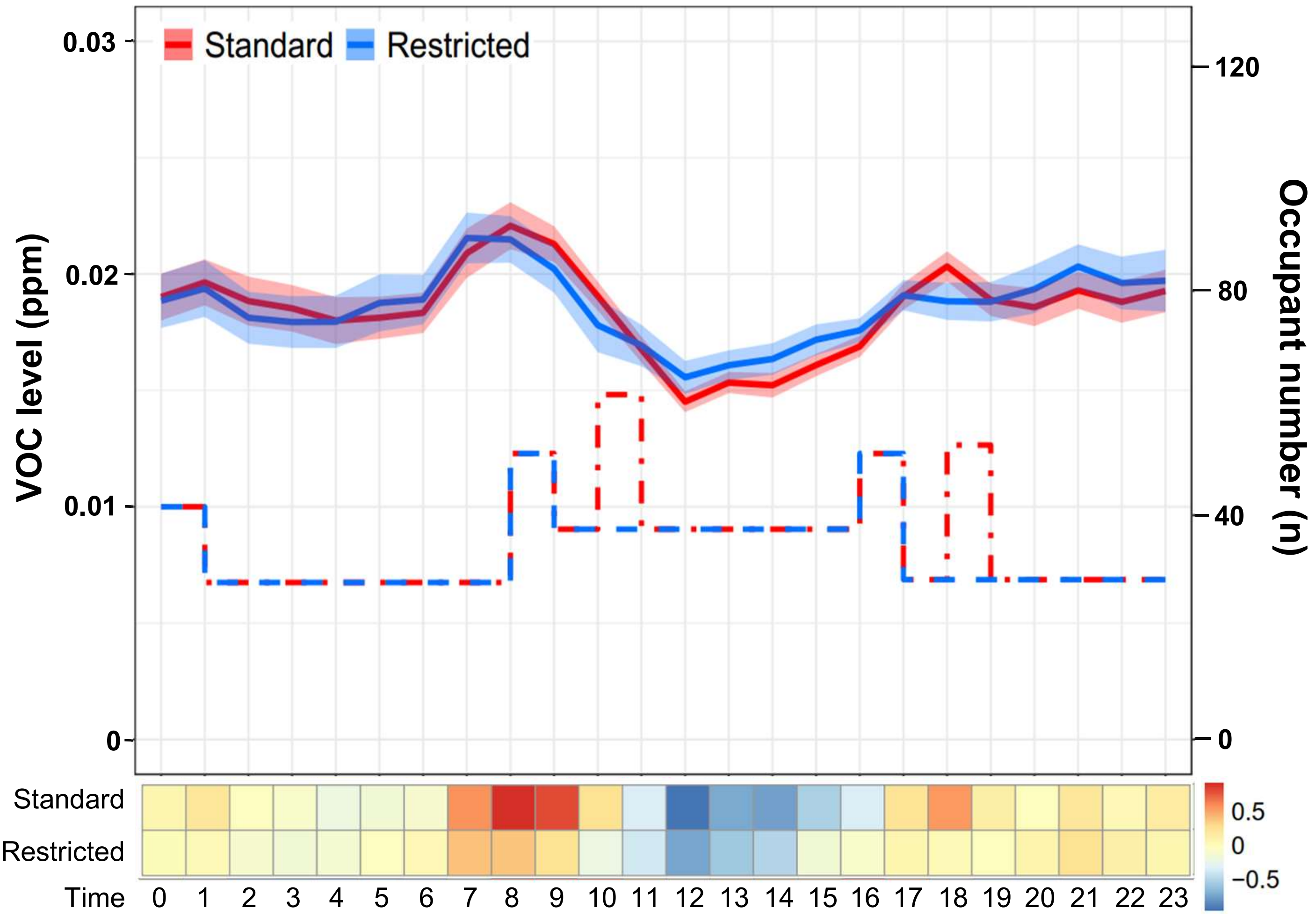

Supplementary Figure 2. Daily temporal variation in VOC levels during restricted visitation versus standard visitation. The line chart and Z-score heat map depict the change in daily VOC concentrations during restricted visitation versus standard visitation. The line chart demonstrates hourly VOC levels in mean and standard deviation. The step plot represents the estimated hourly occupant numbers during standard visitation (red dot-dash line) and restricted visitation (blue dash line). The Z-score in the heat map is transformed based on the mean and standard deviation in each group.

Characteristics	Area A		Area B	
	Restricted	Standard	Restricted	Standard
**Descriptive data**				
n	70,560	73,440	70,560	73,440
Mean (SD)	1.35 (2.73)	3.09 (2.76)	2.13 (3.90)	2.42 (6.44)
Minimum	0	0	0	0
Median	1	2	1	2
IQR	(0, 1)	(1, 4)	(0, 2)	(1, 3)
Maximum	101	82	58	221

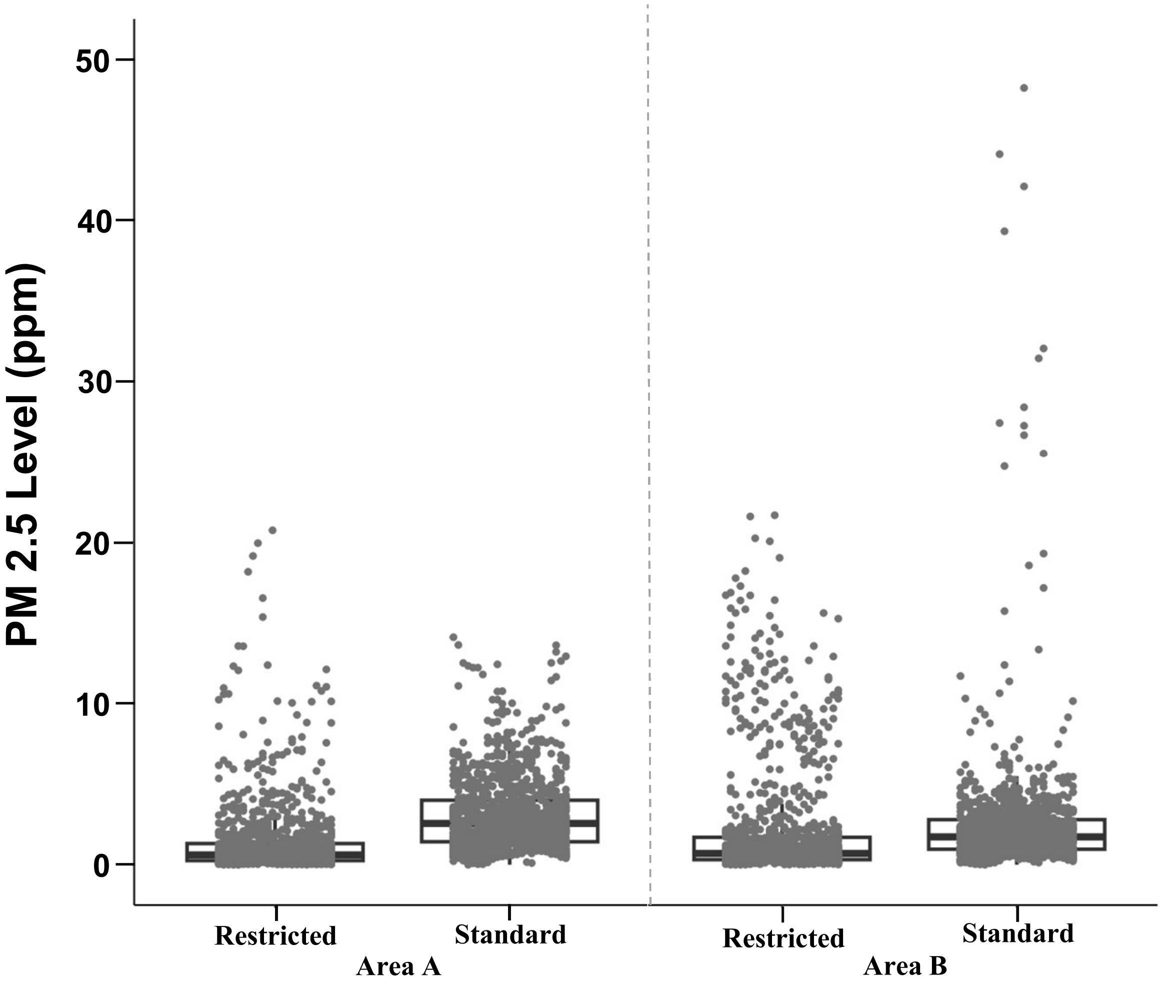

Supplementary Figure 3. Descriptive data and boxplots of PM2.5 concentrations during restricted visitation versus standard visitation in area A (left) and area B (right) of SICU1. SD: standard deviation, IQR: interquartile range. Each point in the figure represents the mean value of hourly data.
